# Controlled Release of Interleukin-1 Receptor Antagonist from Hyaluronic Acid-Chitosan Microspheres Attenuates Interleukin-1*β*-Induced Inflammation and Apoptosis in Chondrocytes

**DOI:** 10.1155/2016/6290957

**Published:** 2016-10-30

**Authors:** Bo Qiu, Ming Gong, Qi-Ting He, Pang-Hu Zhou

**Affiliations:** Department of Orthopedics, Renmin Hospital of Wuhan University, No. 238, Liberation Road, Hubei, Wuhan 430060, China

## Abstract

This paper investigates the protective effect of interleukin-1 receptor antagonist (IL-1Ra) released from hyaluronic acid chitosan (HA-CS) microspheres in a controlled manner on IL-1*β*-induced inflammation and apoptosis in chondrocytes. The IL-1Ra release kinetics was characterized by an initial burst release, which was reduced to a linear release over eight days. Chondrocytes were stimulated with 10 ng/ml IL-1*β* and subsequently incubated with HA-CS-IL-1Ra microspheres. The cell viability was decreased by IL-1*β*, which was attenuated by HA-CS-IL-1Ra microspheres as indicated by an MTT assay. ELISA showed that HA-CS-IL-1Ra microspheres inhibited IL-1*β*-induced inflammation by attenuating increases in NO_2_^−^ and prostaglandin E2 levels as well as increase in glycosaminoglycan release. A terminal deoxyribonucleotide transferase deoxyuridine triphosphate nick-end labeling assay revealed that the IL-1*β*-induced chondrocyte apoptosis was decreased by HA-CS-IL-1Ra microspheres. Moreover, HA-CS-IL-1Ra microspheres blocked IL-1*β*-induced chondrocyte apoptosis by increasing B-cell lymphoma 2 (Bcl-2) and decreasing Bcl-2-associated X protein and caspase-3 expressions at mRNA and protein levels, as indicated by reverse-transcription quantitative polymerase chain reaction and western blot analysis, respectively. The results of the present study indicated that HA-CS-IL-1Ra microspheres as a controlled release system of IL-1Ra possess potential anti-inflammatory and antiapoptotic properties in rat chondrocytes due to their ability to regulate inflammatory factors and apoptosis associated genes.

## 1. Introduction

Osteoarthritis (OA) is an age-related degenerative disease of the joints leading to progressive cartilage damage [1]. Inflammatory cytokines and mediators trigger the underlying pathological mechanism of OA by leading to the production of various proteases which disrupt cell matrix signaling and result in the depletion of collagen and proteoglycan [2]. Interleukin-1*β* (IL-1*β*) is one of the most important inflammatory cytokines and has a central role in the pathogenesis of OA [3]. IL-1*β* has been reported to accelerate the aging and increase the chondrocyte apoptosis which is thought to be important in OA [4–6]. Consequently, the inhibition of the IL-1*β* pathway presents a promising means of preventing inflammation and apoptosis in chondrocytes in the pathogenesis of OA. One of the major endogenous inhibitors of the IL-1 pathway is the anti-inflammatory cytokine interleukin-1 receptor antagonist (IL-1Ra) which binds the IL-1RI with similar specificity and affinity to that of IL-1, while it does not activate any downstream signals [7, 8]. Of note, IL-1Ra has been demonstrated to inhibit the progression of OA, suggesting that IL-1Ra is a suitable target for the treatment of OA [9, 10].

Hyaluronic acid (HA) is a naturally occurring glycosaminoglycan and a component of cartilage matrix and synovial fluid [11]. HA possesses anabolic, analgesic, anti-inflammatory, and chondroprotective activities [12]. In OA, intra-articular injection of HA was shown to augment the flow of joint fluid, improve the viscoelasticity of synovial fluid, normalize endogenous hyaluronate synthesis, reduce pain, inhibit hyaluronate degradation, and improve the range of motion in the knee [13, 14]. A previous study by our group has demonstrated that HA dose-dependently suppressed chondrocyte apoptosis in a model of IL-1*β*-induced OA [15].

Chitosan (CS), an abundant polysaccharide, is obtained by deacetylation of its precursor polymer chitin [16]. It features excellent biocompatibility and biodegradability alongside ecological safety as well as low toxicity and immunogenicity. CS possesses versatile biological activities and applications, including antimicrobial effect and use as a scaffold in tissue engineering and a carrying system for the delivery of drugs and nucleotides [17–21]. Injection of CS solution into the murine knee joint caused a significant increase in the density of newly formed chondrocytes, suggesting that it could facilitate the healing of the cartilage. CS promotes attachment, proliferation, and viability of mesenchymal stem cells, and, thanks to these promising features, CS and its derivatives are considered as very interesting biomaterials [22].

The main problem for the treatment of OA is that anything injected into a joint tends to come back out again very quickly, which restricts the effectiveness of intra-articular treatments to acute conditions. There is a pressing need for technologies to retain drugs in joints and thus permit sustained therapeutic responses in OA [23]. Therefore, the present study attempted to combine the virtues of HA and CS to develop IL-1Ra-carrying microspheres and investigated their effect against IL-1*β*-stimulated inflammation and apoptosis in rat chondrocytes in an in vitro model of OA. It indicated that HA-CS-IL-1Ra microspheres efficiently released IL-1Ra to attenuate inflammation and apoptosis in chondrocytes and may be suitable for the treatment of OA.

## 2. Materials and Methods

### 2.1. Materials

CS (molecular weight, 150 kDa; deacetylation: 98%), HA (molecular weight, 500–730 kDa), and sodium tripolyphosphate (STPP) were provided by Sigma-Aldrich (St. Louis, MO, USA). Recombinant rat IL-1*β* and IL-1Ra were purchased from PeproTech (Rocky Hill, NJ, USA). Trypsinase, collagenase II, Dulbecco's modified Eagle's medium (DMEM)/F12, foetal bovine serum (FBS), 3-(4,5-dimethylthiazol-2-yl)-2,5-diphenyltetrazolium bromide (MTT), 6-diamidino-2-phenylindole dihydrochloride (DAPI), and penicillin/streptomycin were obtained from Gibco (Thermo Fisher Scientific, Waltham, MA, USA). Rabbit monoclonal antibody (IgG) for Bcl-2-associated X protein (Bax, Cat. number 14796) and rabbit polyclonal antibodies (IgG) for B-cell lymphoma 2 (Bcl-2, Cat. number 2876) and caspase-3 (Cat. number 9662) were purchased from Cell Signal Technology (Beverly, MA, USA). An in situ cell apoptosis detection kit was purchased from Roche Diagnostics (Cat. number 11684795910, Basel, Switzerland). All other chemicals used in this study were of analytical grade and obtained from Sigma-Aldrich (St. Louis, MO, USA) unless otherwise stated.

### 2.2. Microsphere Preparation and Characterization

HA-CS microspheres were prepared according to an ionic cross-linking method in emulsion according to previously described procedures with certain modifications [24]. Briefly, 2 g of CS was dispersed into the acetic acid (100 mL) under vigorous stirring for 3 h at ambient temperature (<20°C) to obtain transparent chitosan emulsion (2% w/v) and HA emulsion (0.1%, w/v) was obtained using an identical method. Subsequently, 10 mL of the CS emulsion and 5 mL of the HA emulsion were immediately mixed with vigorous stirring to obtain stable HA-CS suspension. Well-mixed suspension of 1 g Span 80 in 100 mL paraffin oil (0.827–0.890 g/mL at 20°C, flash point at 215°C) was placed in a 200 mL beaker and stirred with a thermostatic magnetic stirrer (MYP11-2, Shanghai, China) at 800 ×g for 1 h. Subsequently, 6 mL of the prepared HA-CS suspension was added to the Span 80 suspension in a dropwise manner at 1 mL/min. The reaction mixture was stirred at the identical speed and temperature to those mentioned above for additional 2 h. Subsequently, 10 mL of STPP solution (10% w/v) was added and the reaction was maintained under identical conditions for 1 h. Following removal of the supernatant (paraffin), HA-CS microspheres at the bottom of the vessel were collected. The microspheres were washed with 10 mL ethanol and 10 mL acetone two times to completely remove the residual paraffin oil and Span 80. Under magnetic stirring at room temperature, 3.5 mL of mixture of an aqueous solution of STPP (0.06 mg/mL) and IL-1Ra was added to 3.5 mL of CS solution (1%, w/v, pH 5.0) under magnetic stirring at room temperature for 10 min for complete stabilization of the system. Next, the microspheres were transferred into Eppendorf tubes and isolated by centrifugation in a glycerol bed at 16,000 ×g for 30 min at 25°C. Supernatant was collected and the microspheres were then resuspended into ultrapure water by shaking on a vortex mixer. Next, the microspheres were centrifuged from the fixed volume of microsphere suspension at 16,000 ×g for 30 min at 25°C without a glycerol bed. The supernatant was discarded and HA-CS-IL-1Ra microspheres were prepared. CS-IL-1Ra microspheres were then prepared using an identical method without HA. Finally, the microspheres were freeze-dried. The sizes and shapes of the microspheres were examined under a scanning electron microscope (SEM, S-800, Hitachi, Tokyo, Japan).

### 2.3. Determination of IL-1Ra Content in CS-IL-1Ra and HA-CS-IL-1Ra Microspheres

The encapsulation efficiency (EE) in CS-IL-1Ra or HA-CS-IL-1Ra microspheres was measured using a microplate reader (Bio-Rad 680, Hercules, CA, USA) at 450 nm wavelength. Briefly, IL-1Ra stock solution was diluted by the supernatant after microsphere reaction solution centrifugation; then the linear relationship between the absorbance and concentration of IL-1Ra was determined at 450 nm wavelength. The suspension containing CS-IL-1Ra or HA-CS-IL-1Ra microspheres was centrifuged at 12,000 ×g for 30 min at 4°C. The supernatant absorbance was firstly determined by microplate reader, and then the amount of free IL-1Ra in the supernatant was calculated according to the standard curve equation. The EE of IL-1Ra in CS-IL-1Ra or HA-CS-IL-1Ra microspheres was calculated using the following equation: EE  (%) = [(total  IL-1Ra − free  IL-1Ra)/total  IL-1Ra] × 100%.

### 2.4. In Vitro Release Profiles

Microspheres (~25 mg) containing IL-1Ra were placed in 1.5 mL microcentrifuge tubes containing 1 mL phosphate-buffered saline (PBS) and 10^4^ U/mL of lysozyme. This suspension was agitated in a water bath at 60 ×g for various time periods of up to fifteen days (0, 1 d, 2 d, 3 d, 4 d, 5 d, 6 d, 7 d, 8 d, 9 d, 10 d, 11 d, 12 d, 13 d, 14 d, and 15 d) at 37°C. Periodically, the microsphere suspension was centrifuged to collect the supernatant for analysis of released IL-1Ra, followed by reresuspension of the microspheres in fresh PBS containing lysozyme. The IL-1Ra concentration in the supernatants was assessed using an ELISA kit (Cat. number MRA00, R&D Systems, Minneapolis, MN, USA) according to the manufacturer's instructions.

### 2.5. Chondrocyte Isolation and Culture

A total of 30 seven-day-old male Sprague-Dawley rats were obtained from the Experimental Animal Center of Wuhan University (Wuhan, China). Rats were euthanized with isoflurane followed by cervical dislocation. The protocols for the animal experiment of the present study were in accordance with the recommendations and guidelines of the National Institutes of Health (Bethesda, MD, USA) and were approved by the Wuhan University Animal Care and Use Committee (Wuhan, China). For the isolation of chondrocytes, cartilage was obtained from the knee joints of the rats and placed into PBS. In brief, the cartilage was minced into small pieces and incubated in a 0.2% trypsin-containing solution with agitation for 2 h at 37°C. Following washing with DMEM and PBS twice, respectively, the cartilage was incubated in 0.2% collagenase with agitation at 37°C overnight. After digestion, the isolated chondrocytes were washed with DMEM and PBS twice, respectively, and then suspended in DMEM/F12 medium supplemented with 10% FBS and 1% antibiotics at 37°C in a humidified atmosphere with 5% CO_2_. Cells were used at passage 0 to 1 to avoid dedifferentiation and maintained in a monolayer culture throughout the study. Cell viability was determined using a cell viability analyzer (viability > 90%, Beckman Coulter, Miami, FL, USA).

## 3. Treatments

First-generation rat chondrocytes were cultured in DMEM/F12 with 2% FBS for 24 h after washing three times with PBS to avoid the influence of any other cytokines. Next, the culture medium was changed to DMEM/F12 in the presence of 10% FBS. Subsequently, IL-1*β* (10 ng/mL) was added to the culture medium followed by incubation for an additional 48 h. Finally, chondrocytes were then cocultured with CS, HA-CS, CS-IL-1Ra, or HA-CS-IL-1Ra microspheres for a period of 4 h. A blank group was kept untreated except for replacement of the medium. A control group consisted of cells treated with 10 ng/mL IL-1*β* alone. Each group consisted of five independent samples from different rats and each experiment was repeated ten times.

### 3.1. Cell Viability Assay

The effect of microspheres on the viability of the chondrocytes was assessed using an MTT assay. Chondrocytes were cultured in 96-well plates at a density of 1 × 10^4^ cells/well for a total volume of 200 *μ*L of the growth medium (DMEM-F12 containing 2% FBS). After 48 h of coculture, the microsphere suspension was discarded and fresh DMEM containing 0.5 mg/mL MTT was added to the chondrocytes followed by incubation at 37°C with 5% CO_2_ for 4 h. The supernatant was removed and 150 *μ*L of dimethylsulfoxide was added. The absorbance at 570 nm was measured using a microplate reader (Bio-Rad 680, Hercules, CA, USA). Wells containing culture medium only were used as a blank control.

### 3.2. Quantification of Nitrite (NO_2_^−^), Prostaglandin E2 (PGE2), and Glycosaminoglycan (GAG) Concentrations

The levels of NO_2_^−^ (Cat. number KGE001, R&D Systems, Minneapolis, MN, USA), PGE2 (Cat. number KGE004B, R&D Systems, Minneapolis, MN, USA), and GAG (Cat. number SBJ-R0791, Nanjing SenBeiJia Biological Technology Co., Nanjing, China) were measured using ELISA kits according to the manufacturers' instructions. The NO_2_^−^ concentration was determined using the respective standard curve and normalized against the control concentration.

### 3.3. Detection of Apoptosis

According to the manufacturer's instructions, the terminal deoxynucleotidyl transferase deoxyuridine triphosphate-biotin nick-end labeling (TUNEL) assay was performed to detect cell apoptosis. Chondrocytes were seeded on cover slips in 24-well plates at a density of 1.25 × 10^5^ cells/well for a total volume of 1 mL of the growth medium (DMEM-F12 containing 2% FBS). They were stained with DAPI at 37°C for 30 min and apoptotic chondrocytes were recognized using dual TUNEL and DAPI staining. Images were randomly selected from three fields of each specimen and the stained cells were counted under ×200 magnification. For each experimental group, three images were randomly selected using an inverted fluorescence microscope (Olympus, Tokyo, Japan).

### 3.4. RNA Extraction and Reverse-Transcription Quantitative Polymerase Chain Reaction (RT-qPCR)

TRIzol and chloroform reagents were used to extract total RNA from chondrocytes according to the manufacturer's instructions. Briefly, after 2 mL of TRIzol reagent was added to split chondrocytes for 20 min, the sample was transferred to 2 mL Eppendorf tube. Subsequently, 400 *μ*L of chloroform was added. The tube was shaken vigorously for 30 s and allowed to stand for 15 min. The sample was then centrifuged at 13,000 ×g for 15 min at 4°C. The supernatant from the final extraction step was transferred to a clean 2 mL Eppendorf tube and the RNA precipitated with 500 *μ*L isopropanol at −20°C for 2 h. Precipitated RNA was collected by centrifugation at 13,000 ×g for 15 min at 4°C; the pellet was washed with 1 mL of 75% ice-cold ethanol. The RNA pellet was resuspended in 20 *μ*L of nuclease-free water and the two duplicate tubes were combined. RNA concentration was measured using a Spectrophotometer (Biolab ND-1000, Thermo Fisher Scientific, Scoresby, VIC, Australia) at 260 nm. RNA purity was assessed by determining the A260/A280 ratio. Purified RNA with an A260/A280 ratio between 1.7 and 2.0 was used in this study. Complimentary DNA (cDNA) was synthesized from RNA using reverse transcriptase and a PrimeScript reverse transcriptase kit (Cat. number AB-1455/A, Fermentas, Thermo Fisher Scientific, Waltham, MA, USA). Quantitative real time PCR (qRT-PCR) was performed using a 20 *μ*L reaction volume containing 10 *μ*L of SYBR® Premix Ex Taq™ II (Takara, Otsu, Japan), 0.4 *μ*L of ROX Reference Dye II, 0.8 *μ*L of forward and reverse primer each, 2 *μ*L of cDNA, and 6 *μ*L of nuclease-free water. Reactions were run on a 7500 Real Time PCR system (Thermo Fisher Scientific, Waltham, MA, USA) for 45 cycles at 95°C for 15 s followed by 60°C for 1 min. Specific PCR products were confirmed by melting-curve analysis and *β*-actin served as an internal control. Gene expression levels were standardized against *β*-actin. The primers used (Takara, Otsu, Japan) are listed in Table 1.

### 3.5. Western Blot Analysis

Proteins were extracted from harvested chondrocytes and protein concentrations were determined using the bicinchoninic acid protein assay kit (Cat. number 23225, Fermentas, Thermo Fisher Scientific, Waltham, MA USA). Every well in the 10% separating SDS-PAGE was loaded with 20 *μ*L protein. Equal quantities of protein were separated using SDS-PAGE and transferred to nitrocellulose membranes (Fermentas, Thermo Fisher Scientific, Waltham, MA USA). The membranes were blocked in PBS containing 5% nonfat dry milk and initially incubated with anti-Bcl-2 which does not cross-react with Bcl-2 beta or other Bcl-2 family members (dilution, 1 : 1000), anti-Bax which recognizes endogenous levels of total Bax protein in rodent samples (dilution, 1 : 1000), or anti-caspase-3 which detects endogenous levels of caspase-3 resulting from cleavage (dilution, 1 : 1000) overnight at 4°C. Subsequently, the membranes were washed three times with TBST (10 mM Tris-HCl, pH 7.4, 100 mM NaCl, 0.2% Tween-20) and incubated with horseradish peroxidase-conjugated secondary antibodies (goat anti-rabbit immunoglobulin G, Cat. number 7075, Cell Signal Technology, Beverly, MA, USA), followed by visualization using an enhanced chemiluminescence kit (Cat. number 35085, Fermentas, Thermo Fisher Scientific, Waltham, MA, USA). Blots were scanned using a gel imaging system (GelDoc-It 310, UVP Co., Upland, CA, USA) and densitometric analyses were performed using Image Lab 4.1 software (Bio-Rad Laboratories, Hercules, CA, USA).

### 3.6. Statistical Analysis

Values are expressed as the mean ± standard deviation and statistical analyses were performed using SPSS software, version 19.0 (International Business Machines, Armonk, NY, USA). Each experimental condition was performed in triplicate wells, and replicates from each culture were averaged and combined as one value for analysis. Significant differences among the mean values of multiple groups were evaluated by analysis of variance followed by the Student-Newman-Keuls method. *P* < 0.05 was considered to indicate a statistically significant difference between values.

## 4. Results

**Figure 1:**
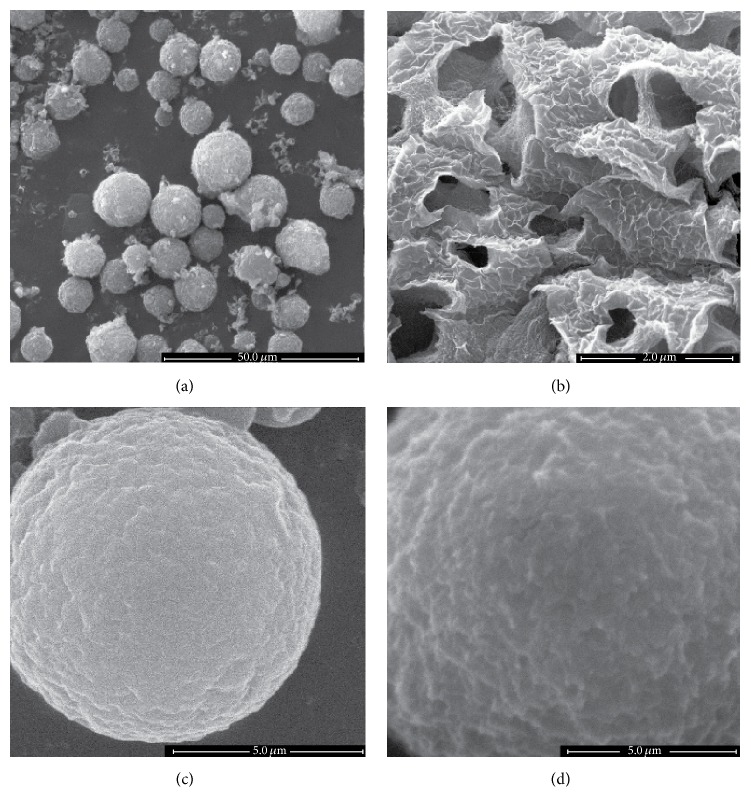
Characterization of microspheres by scanning electron microscopy. (a) Microspheres were spherical and ranged in size from 7 to 16 *μ*m. (b) The microsphere surface appeared to be loose and porous, and the internal structure was cell-like. With regard to the variation in composition and structure, there was a slight increase in the microsphere size from (c) CS-IL-1Ra to (d) HA-CS-IL-1Ra microspheres (scale bar, 5 *μ*m). IL-1Ra: interleukin-1 receptor antagonist; HA: hyaluronic acid; CS: chitosan.

Characterization of microspheres: the microsphere morphology was observed by SEM (Figure 1). The HA-CS-IL-1Ra microspheres, fabricated using the emulsification method in the presence of STPP, were spherical in shape with smooth surface. The resulting microspheres were spherical and ranged in size from 7 to 16 *μ*m (Figure 1(a)). The microsphere surface appeared to be loose and porous, and the internal structure was cell-like (Figure 1(b)). With regard to the variation in composition and structure, a slight increase in the microsphere size was observed from CS-IL-1Ra (Figure 1(c)) to HA-CS-IL-1Ra microspheres (Figure 1(d)). It was evident that the surface of the microspheres containing HA was more porous than that of the microspheres void of HA.

The EE of IL-1Ra in CS-IL-1Ra or HA-CS-IL-1Ra microspheres were 52.6 ± 6.8% and 65.3 ± 8.1%, respectively. The EE of IL-1Ra in HA-CS-IL-1Ra microspheres was significantly higher than that of CS-IL-1Ra microspheres (*P* < 0.05).

**Figure 2:**
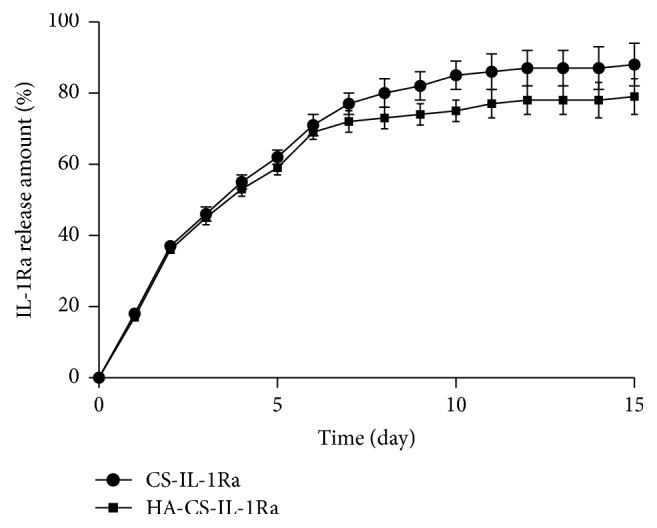
Release kinetics of HA-CS-IL-1Ra and CS-IL-1Ra microspheres. Values were expressed as the mean ± standard deviation. IL-1Ra: interleukin-1 receptor antagonist; HA: hyaluronic acid; CS: chitosan.

In vitro release profiles: Figure 2 shows the release kinetics of IL-1Ra from both types of microspheres. IL-1Ra release kinetics was monitored over 15 days and was characterized by an initial burst release, which was gradually reduced to a linear release. The release rate of IL-1Ra in the HA-CS-IL-1Ra group was slower than that in the CS-IL-1Ra group. The final release rate was ~85% in the CS-IL-1Ra group within 10 days of incubation, while it was ~75% in the HA-CS-IL-1Ra group within the same period. The release rate of IL-1Ra in the HA-CS-IL-1Ra group was significantly lower than that of IL-1Ra in the CS-IL-1Ra group within 10 days of incubation (*P* < 0.05). In conclusion, compared with that in CS-IL-1Ra group, IL-1Ra was released in a slower and more continuous manner in the HA-CS-IL-1Ra group.

**Figure 3:**
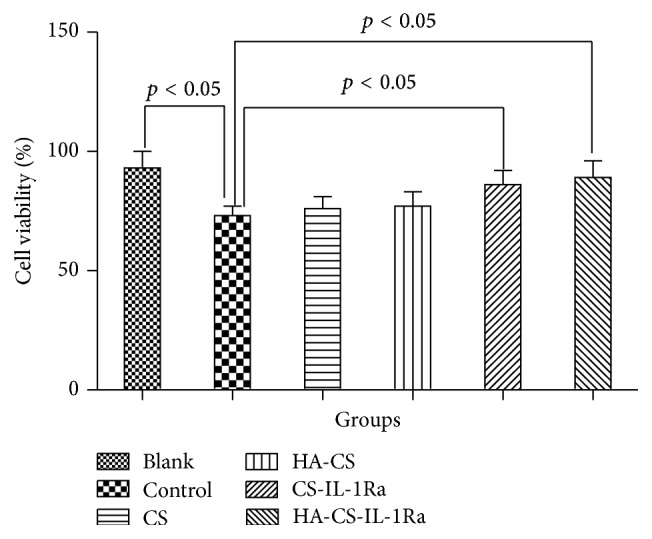
HA-CS-mediated release of IL-1RA attenuated IL-1*β*-induced reduction of chondrocyte viability. Values were expressed as the mean ± standard deviation. IL-1Ra: interleukin-1 receptor antagonist; HA: hyaluronic acid; CS: chitosan; AI: apoptotic index.

IL-1Ra-releasing microspheres attenuate IL-1*β*-mediated reduction in cell viability.  Figure 3 shows the cell viability in the various treatment groups. The cell viability of control group was 73 ± 4%, which was significantly lower than 93 ± 7% of blank group (*P* < 0.05). No significant differences in cell viability among the control (73 ± 4%), CS group (76 ± 5%), and HA-CS group (77 ± 6%) were observed. The cell viability of CS or HA-CS group was slightly but not significantly higher than that of control group. However, cotreatment with HA-CS-IL-1Ra or CS-IL-1Ra microspheres significantly increased the cell viability to 89 ± 7 and 86 ± 6%, respectively (*P* < 0.05 versus control).

**Figure 4:**
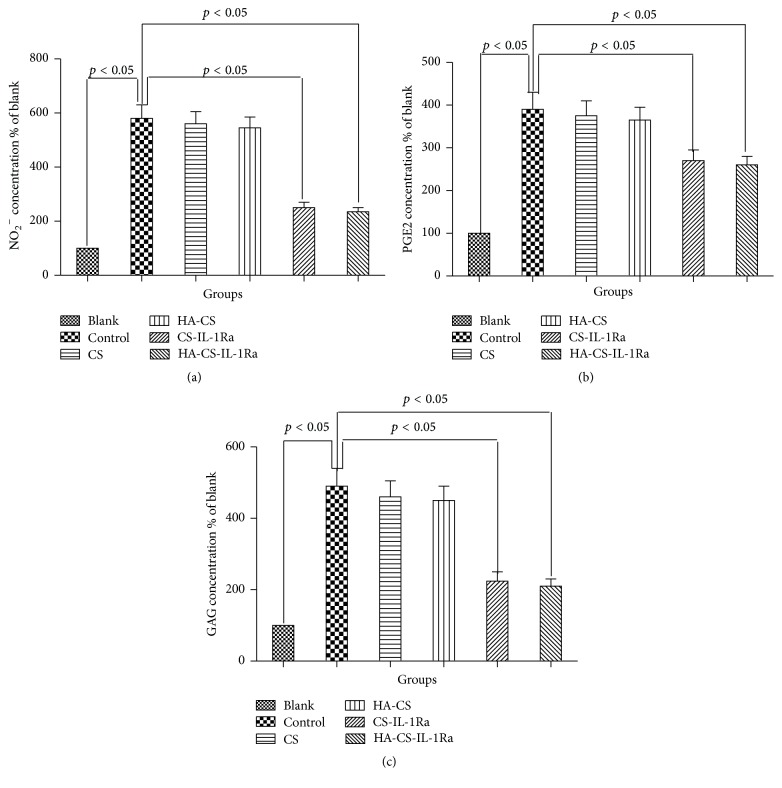
Effect of microspheres on IL-1*β*-induced NO_2_^−^, PGE2, and GAG production. Values were expressed as the mean ± standard deviation. IL-1Ra: interleukin-1 receptor antagonist; HA: hyaluronic acid; CS: chitosan; PGE2: prostaglandin E2; GAG: glycosaminoglycan.

IL-1Ra-releasing microspheres attenuated IL-1*β*-induced release of NO_2_^−^, PGE2, and GAG by chondrocytes.  Figure 4 shows the concentrations of NO_2_^−^, PGE2, and GAG in the various experimental groups. Stimulation with IL-1*β* (10 ng/mL) alone led to a 5.8-fold increase in NO_2_^−^ production in the supernatant (*P* < 0.05). CS and HA-CS microspheres slightly but not significantly decreased NO_2_^−^ production (*P* > 0.05), whereas CS-IL-1Ra and HA-CS-IL-1Ra microspheres significantly decreased the NO_2_^−^ production (*P* < 0.05). Similar effect was observed for the PGE2 and GAG concentrations in the supernatant, which was significantly attenuated by CS-IL-1Ra and HA-CS-IL-1Ra microspheres (*P* < 0.05 versus control).

**Figure 5:**
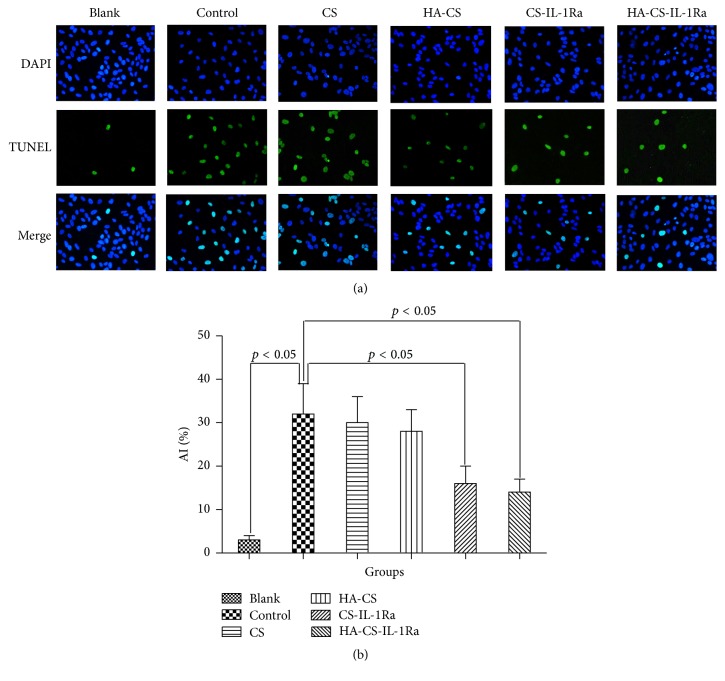
HA-CS-mediated release of IL-1Ra reduces IL-1*β*-induced chondrocyte apoptosis. (a) Apoptosis in various treatment groups was assessed by TUNEL staining and fluorescence microscopic analysis (magnification, ×200). Nuclei were counterstained with DAPI. (b) AIs were obtained by quantification of (a). Values were expressed as the mean ± standard deviation. IL-1Ra: interleukin-1 receptor antagonist; HA: hyaluronic acid; CS: chitosan; AI: apoptotic index; TUNEL: terminal deoxyribonucleotide transferase dUTP nick-end labeling.

IL-1Ra-releasing microspheres reduced IL-1*β*-induced chondrocyte apoptosis.  Figure 5 shows TUNEL staining of chondrocytes in the different treatment groups for the apoptosis detection. The percentage of TUNEL-positive cells in the control group was 37 ± 6%, while that in the blank group was significantly lower at only 3 ± 1% (*P* < 0.05). Compared with the control group, cotreatment with CS or HA-CS microspheres slightly but not significantly decreased the apoptotic rate of chondrocytes (37 ± 6 versus 35 ± 5 and 34 ± 4%, resp.). However, following coculture with CS-IL-1Ra or HA-CS-IL-1Ra microspheres, the percentages of TUNEL-positive cells were 23 ± 4 or 21 ± 3%, respectively, which were significantly lower than those in the control group (*P* < 0.05).

**Figure 6:**
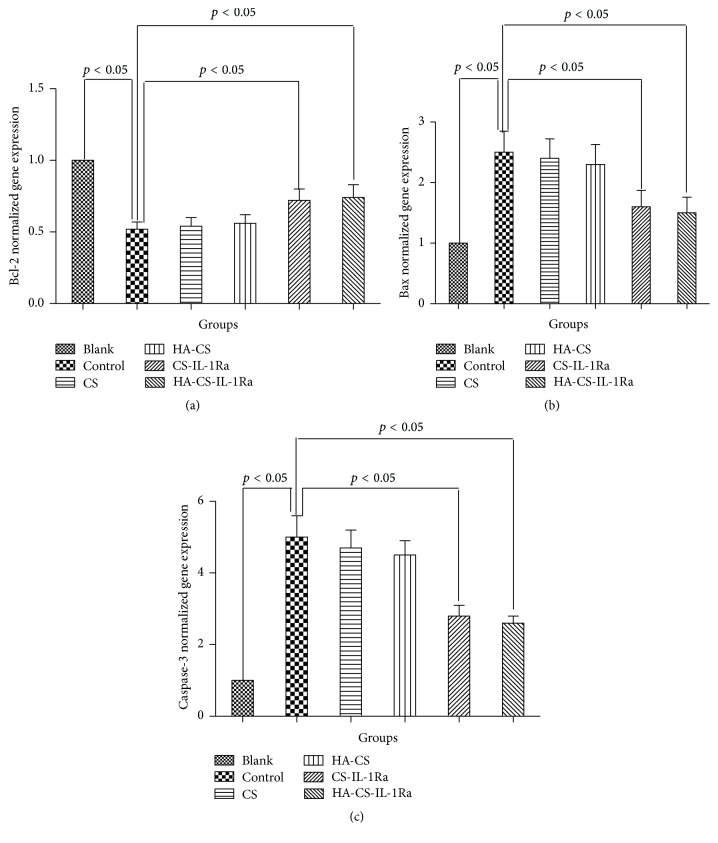
Effect of microspheres on the relative expressions of Bcl-2, Bax, and caspase-3 mRNA. Expression levels were normalized to *β*-actin. Values were expressed as the mean ± standard deviation. IL-1Ra: interleukin-1 receptor antagonist; HA: hyaluronic acid; CS: chitosan.

IL-1Ra-releasing microspheres attenuated IL-1*β*-induced apoptosis signaling.  Figure 6 shows the gene expressions of Bcl-2, Bax, and caspase-3 in various experimental groups. Stimulation with IL-1*β* (10 ng/mL) led to a significant decrease in Bcl-2 expression but significant increases in Bax and caspase-3 expressions compared with those in the blank group (*P* < 0.05). No significant differences in the gene expressions of Bcl-2, Bax, and caspase-3 were found among the control, CS, and HA-CS groups (*P* > 0.05). The gene expressions of Bcl-2, Bax, and caspase-3 in CS or HA-CS groups were slightly but not significantly changed compared to those in the control group. However, the Bcl-2 expression was significantly increased after cotreatment with CS-IL-1Ra or HA-CS-IL-1Ra microspheres (*P* < 0.05), whereas the expressions of Bax and caspase-3 were significantly decreased (*P* < 0.05) after cotreatment with CS-IL-1Ra or HA-CS-IL-1Ra microspheres compared with those in the control group (*P* < 0.05). However, there were no significant differences in the gene expressions of Bcl-2, Bax, and caspase-3 between the CS-IL-1Ra and HA-CS-IL-1Ra groups.

**Figure 7:**
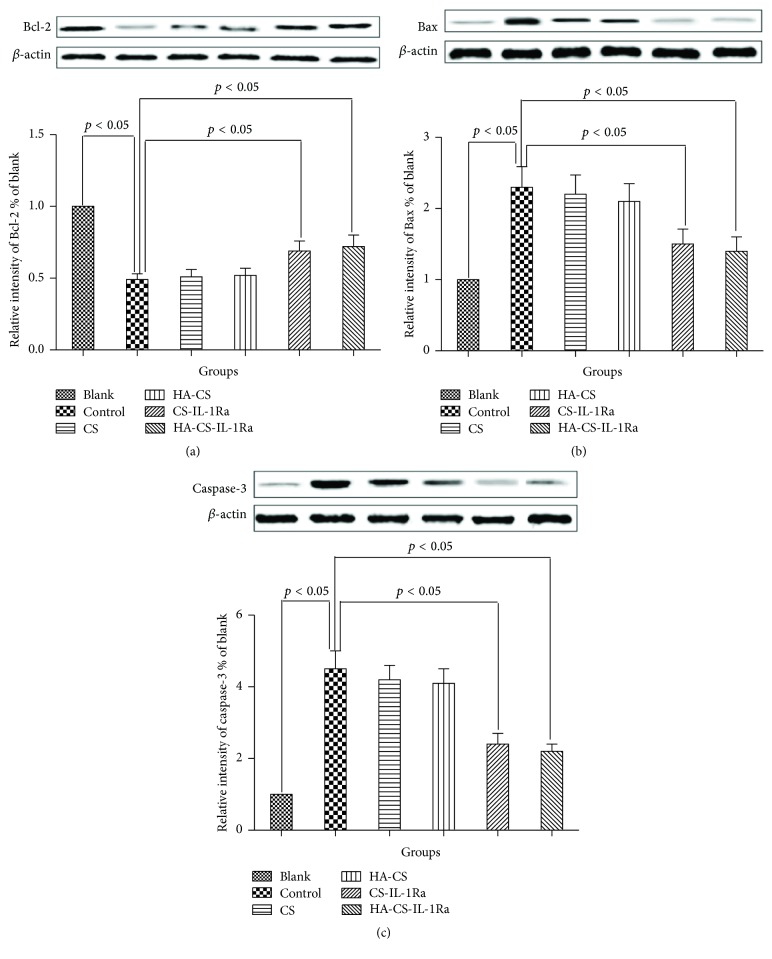
Western blot analysis of Bcl-2, Bax, and caspase-3. Lanes: 1, blank; 2, control; 3, CS; 4, HA-CS; 5, CS-IL-1Ra; 6, HA-CS-IL-1Ra. Protein levels were normalized to *β*-actin. Values were expressed as the mean ± standard deviation. IL-1Ra: interleukin-1 receptor antagonist; HA: hyaluronic acid; CS: chitosan; Bcl-2: B-cell lymphoma 2; Bax: Bcl-2-associated X protein.

Figure 7 shows the protein levels of Bcl-2, Bax, and caspase-3 RT-PCR for Bcl-2, Bax, and caspase-3 in the experimental groups, revealing trends similar to the mRNA levels of the respective proteins. No significant differences were observed in the expressions of Bcl-2, Bax, and caspase-3 among the control, CS, and HA-CS groups. The protein levels of Bcl-2, Bax, and caspase-3 in CS or HA-CS group were slightly but not significantly changed compared to those in the control group. However, the expression of Bcl-2 was significantly increased in the CS-IL-1Ra and HA-CS-IL-1Ra groups compared with that in the control group (*P* < 0.05), while the expressions of Bax and caspase-3 were significantly decreased (*P* < 0.05). However, there were no significant differences in the gene expressions of Bcl-2, Bax, and caspase-3 between the CS-IL-1Ra and HA-CS-IL-1Ra groups.

## 5. Discussion

OA, which is accompanied by joint dysfunction and pain, is the most prevalent type of degenerative joint disease [25]. It is widely accepted that increases in IL-1*β* levels can lead to the production and accumulation of high levels of proinflammatory cytokines and trigger apoptosis in chondrocytes [26]. Of note, sustained release of IL-1Ra from HA-CS microspheres to inhibit the IL-1*β* pathway may prevent inflammation and apoptosis in chondrocytes.

In the present study, one of the main factors affecting the results was the microsphere structure. As the surface of the HA-CS-IL-1Ra microspheres was loose and porous and the internal structure appeared cell-like, it was obvious that HA conjugation enhanced the ability of the microspheres to interact with the chondrocytes and to release the loaded drug in a controlled manner. Furthermore, the release rate of IL-1Ra from the HA-CS-IL-1Ra microspheres was slower than that of CS-IL-1Ra microspheres. This result further confirmed that release kinetics of proteins from the microspheres was influenced by the microsphere structure. HA, carrying high negative charges, which allow for an electrostatic interaction with protonated chitosan in an aqueous acidic solution, may have been accountable for the slow drug release.

In the current study, CS or HA-CS slightly but not significantly increased the cell viability of chondrocytes. Furthermore, the percentages of viable cells in the CS-IL-1Ra and HA-CS-IL-1Ra groups were significantly higher than those in the control group, with the percentage of viable cells in the HA-CS-IL-1Ra group being slightly but not significantly higher than that in the CS-IL-1Ra group. These findings are in accordance with those of our previous study [24].

In our paper, we found that CS or HA-CS slightly but not significantly decreased NO_2_^−^, PGE2, and GAG, a finding consistent with previous investigations [27, 28]. The findings of the present study suggested that CS-IL-1Ra and HA-CS-IL-1Ra inhibited the production of inflammatory agents such as NO_2_^−^ and PGE2, while decreasing the degradation of GAG induced by IL-1*β* in chondrocytes. The accumulative production of NO_2_^−^ is due to the upregulated isoform nitric oxide synthase levels due to PGE2, which has an important role in human adult articular cartilage homeostasis and is linked to the pathophysiology of osteoarthritis [29]. GAG is a polysaccharide which together with collagen type II forms the main component of the cartilage matrix. It has been confirmed that secretion of GAG by chondrocytes cultured in vitro can be regarded as an indicator of the maintenance of the chondrocytes phenotype [30]. In the present study, upon stimulation with IL-1*β*, a significant increase was observed in the release of GAG into the culture medium which was considered as an indicator of rapid proteoglycan degradation. The GAG content was rescued in the presence of CS-IL-1Ra and HA-CS-IL-1Ra. This suggests that both of microspheres containing IL-1Ra can contribute to inhibition of proteoglycan degradation, indicating a protective effect on chondrocytes phenotype. Our results are consistent with the previous study which showed rapid proteoglycan degradation in a cartilage-like extracellular matrix [31]. However, slight but not significant differences in the levels of NO_2_^−^, PGE2, and GAG between CS-IL-1Ra and HA-CS-IL-1Ra groups were identified. These findings suggest that IL-1Ra may act as an anti-inflammatory agent similar to nonsteroid anti-inflammatory drugs, which have been shown to ameliorate OA symptoms by inhibiting the production of PGE2. In addition, IL-1Ra may maintain the chondrocyte phenotype. Furthermore, it was demonstrated that the controlled release of IL-1Ra by HA-CS was prolonged compared to that by CS alone, which may have been due to the highly negative charges and the bulky and gel-like characteristics of HA, which may have promoted the controlled release by modifying the bond between CS and the protein [32]. HA simultaneously combined with CS through electrostatic interactions. In the present study, the application of HA as a component of CS microspheres was demonstrated to be a reasonable approach for enhancing the potency of CS-IL-1Ra in the treatment of chondrocytes affected by OA.

Consistent with the results of previous studies, the present study found that IL-1*β* induced apoptosis in chondrocytes [33, 34]. CS or HA-CS slightly but not significantly decreased the apoptotic rate of chondrocytes. This is in good agreement with the previous reports [28, 35]. However, the percentage of apoptotic cells was significantly decreased in chondrocytes cotreated with CS-IL-1Ra or HA-CS-IL-1Ra microspheres. The percentage of apoptotic cells in the HA-CS-IL-1Ra group was slightly but not significantly less than that in the CS-IL-1Ra group. It can be inferred that IL-1Ra can suppress chondrocyte apoptosis in an in vitro model of IL-1*β*-induced OA and that the HA-CS-IL-1Ra microspheres are more potent than CS-IL-1Ra microspheres.

Bcl-2, Bax, and caspase-3 are crucial biomarkers for cell apoptosis, with caspase-3 acting as an apoptotic executor [36, 37]. The present study showed that CS or HA-CS slightly but not significantly changed the gene and protein levels of Bcl-2, Bax, and caspase-3. Our data agree with the previous reports [38, 39]. In addition, the expression of Bcl-2 was significantly increased in the CS-IL-1Ra and HA-CS-IL-1Ra groups, while the expressions of Bax and caspase-3 were decreased at the transcriptional and translational levels compared with those in the control group, with HA-CS-IL-1Ra having slightly more potent effect than CS-IL-1Ra. These results further confirmed the microsphere effect on cell apoptosis and proliferation.

The suppression of inflammation and apoptosis within the joint represents a clinical treatment method for OA, in which microspheres may be employed as efficient drug-loaded carriers. The results of the present study revealed that the sustained release of IL-1Ra from HA-CS- IL-1Ra microspheres prevented IL-1*β*-induced inflammation and apoptosis in chondrocytes. These results suggest that HA-CS microspheres are potent IL-1Ra carriers with controlled and prolonged release properties for the treatment of OA.

## Figures and Tables

**Table 1:** Sequences of primers used for reverse-transcription quantitative polymerase chain reaction.

Gene	Sense	Sequence 5′ → 3′	Size (bp)
Bcl-2	F	CCACCAAGAAAGCAGGAAACC	177
	R	GGCAGGATAGCAGCACAGG	

Bax	F	CAGATGTGGTCTATAATGC	110
	R	CTAATCAAGTCAAGGTCAC	

Caspase-3	F	CATGGAAGCGAATCAATGGACT	139
	R	CTGTACCAGACCGAGATGTCA	

*β*-actin	F	GCAGAAGGAGATCACTGCCCT	136
	R	GCTGATCCACATCTGCTGGAA	

F: forward; R: reverse.
