# Corrigendum to “Controlled Release of Interleukin-1 Receptor Antagonist from Hyaluronic Acid-Chitosan Microspheres Attenuates Interleukin-1*β*-Induced Inflammation and Apoptosis in Chondrocytes”

**DOI:** 10.1155/2020/6942710

**Published:** 2020-09-18

**Authors:** Bo Qiu, Ming Gong, Qi-Ting He, Pang-Hu Zhou

**Affiliations:** Department of Orthopedics, Renmin Hospital of Wuhan University, No. 238, Liberation Road, Wuhan, 430060 Hubei, China

In the article titled “Controlled Release of Interleukin-1 Receptor Antagonist from Hyaluronic Acid-Chitosan Microspheres Attenuates Interleukin-1*β*-Induced Inflammation and Apoptosis in Chondrocytes” [1], there were several errors including the inadvertent reuse of images from the authors' earlier work, errors in the primers, and a lack of a reference to the authors' related article from which they reused wording.

The authors raised to the attention of the journal an error in Figure 1, where the images were incorrectly reused from those in the article titled “Inhibition of interleukin-1beta-stimulated dedifferentiation of chondrocytes via controlled release of CrmA from hyaluronic acid-chitosan microspheres” in *BMC Musculoskeletal Disorders* [2]. The authors sincerely apologize for their carelessness and for this error. The figure should be corrected as follows:

The authors did not discuss their related work in *Molecular Medicine Reports*, from which they reused around 1000 words [3]. That article mainly exploited the controlled release of interleukin-1 receptor antagonist from chitosan microspheres without hyaluronic acid. In this study, the authors investigated the protective effect of interleukin-1 receptor antagonist on IL-1*β*-induced inflammation and apoptosis including MTT assay, NO_2_^−^, prostaglandin E2, B-cell lymphoma 2, Bcl-2-associated X protein, and caspase-3 expressions at mRNA or protein levels; the previous work only studied the effect of inhibition of matrix metalloproteinases induced by IL-1*β*.

The primers for Bcl-2, Bax, Caspase-3, and beta-actin are specific to the human genes (as checked using Primer BLAST and BLASTn), but the cells used in the study are Sprague-Dawley rat chondrocytes. The authors say the incorrect primer information was collected during the writing of the article.  Table 1 should be corrected as follows:

The Western Blots in Figure 7 were adjusted for contrast and exposure, and the original images are available in the supplementary materials.

## Figures and Tables

**Figure 1:**
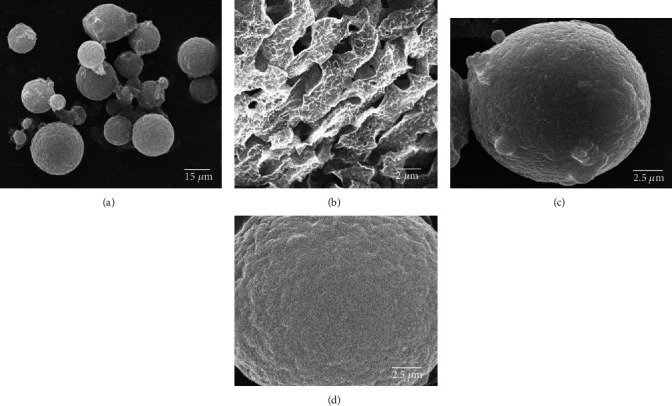
Characterization of microspheres by scanning electron microscopy. (a) Microspheres were spherical and ranged in size from 7 to 16 *μ*m. (b) The microsphere surface appeared to be loose and porous, and the internal structure was cell-like. With regard to the variation in composition and structure, there was a slight increase in the microsphere size from (c) CS-IL-1Ra to (d) HA-CS-IL-1Ra microspheres (scale bar, 5 *μ*m). IL-1Ra: interleukin-1 receptor antagonist; HA: hyaluronic acid; CS: chitosan.

**Table 1:** Sequences of primers used for reverse-transcription quantitative polymerase chain reaction.

Gene	Sense	Sequence 5′→3′
Bcl-2	F	CTTCAGGGATGGGGTGAACT
	R	ATCAAACAGAGGTCGCATGC
Bax	F	GAGACACCTGAGCTGACCTT
	R	CGTCTGCAAACATGTCAGCT
Caspase-3	F	CATGCACATCCTCACTCGTG
	R	CCCACTCCCAGTCATTCCTT
*β*-Actin	F	AGTGCTGTGGGTGTAGGTAC
	R	GCAAAGAGGGCAAGAACACA

## Supplementary Materials

Supplementary materials Original images for the Western Blots in Figure 7 (BMRI 6290957 Figure 7 Western Blots.zip).
