# Supplementary figures and images for: Corrigendum to “Controlled Release of Interleukin-1 Receptor Antagonist from Hyaluronic Acid-Chitosan Microspheres Attenuates Interleukin-1β-Induced Inflammation and Apoptosis in Chondrocytes”
Source: Biomed Res Int. 2020 Sep 18;2020:6942710. doi: 10.1155/2020/6942710 (PMC7533790; doi:10.1155/2020/6942710)

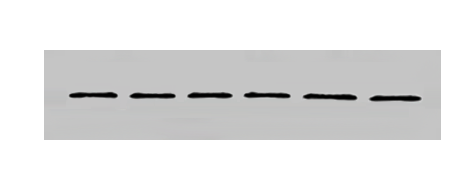

Supplement: Supplementary materials — Original images for the Western Blots in Figure 7 (BMRI 6290957 Figure 7 Western Blots.zip). [file 6942710.f1.zip › Figure-7--¥-actin-1.tif]

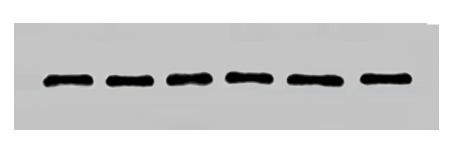

Supplement: Supplementary materials — Original images for the Western Blots in Figure 7 (BMRI 6290957 Figure 7 Western Blots.zip). [file 6942710.f1.zip › Figure-7--¥-actin-2.tif]

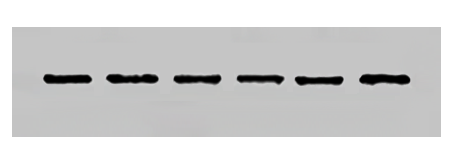

Supplement: Supplementary materials — Original images for the Western Blots in Figure 7 (BMRI 6290957 Figure 7 Western Blots.zip). [file 6942710.f1.zip › Figure-7--¥-actin-3.tif]

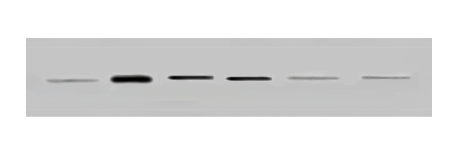

Supplement: Supplementary materials — Original images for the Western Blots in Figure 7 (BMRI 6290957 Figure 7 Western Blots.zip). [file 6942710.f1.zip › Figure-7-Bax.tif]

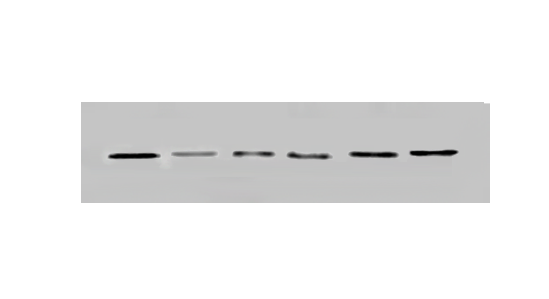

Supplement: Supplementary materials — Original images for the Western Blots in Figure 7 (BMRI 6290957 Figure 7 Western Blots.zip). [file 6942710.f1.zip › Figure-7-Bcl-2.tif]

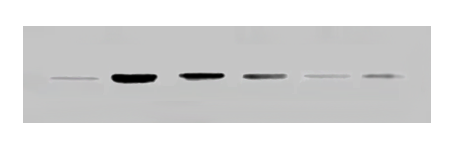

Supplement: Supplementary materials — Original images for the Western Blots in Figure 7 (BMRI 6290957 Figure 7 Western Blots.zip). [file 6942710.f1.zip › Figure-7-Caspase-3.tif]
